# Connecting the dots: internet access, income, and clean cooking energy adoption among rural households in Pakistan

**DOI:** 10.3389/fnut.2026.1779789

**Published:** 2026-07-22

**Authors:** Nawab Khan, Xuanguo Xu

**Affiliations:** 1 College of Economics and Management, Shandong Agricultural University, Tai’an, China; 2 College of Public Administration, Shandong Agricultural University, Tai’an, China

**Keywords:** clean cooking energy, digital connectivity, energy transition, internet use, Pakistan, rural households

## Abstract

Access to clean cooking energy is critical for improving rural health and environmental sustainability. However, adoption remains limited in many developing regions due to financial, infrastructural, and informational constraints. Drawing on innovation diffusion and information-access frameworks, this study examines the relationship between internet use and clean cooking energy adoption among rural households in Pakistan. The analysis is based on survey data from 600 farm households collected in 2025 using a stratified random sampling approach. Employing Logit models, instrumental variable estimation, and mediation analysis, we assess both the direct effect of internet use and the mediating role of household income. Considering the potential endogeneity arising from self-selection and omitted variables, an instrumental variable approach is applied, and the results confirm that the positive association between internet use and clean cooking energy adoption remains robust. The findings show that internet use significantly increases the likelihood of adopting clean cooking energy. Mediation analysis reveals that household income partially mediates this relationship, indicating that digital connectivity promotes clean energy adoption by enhancing both economic capacity and access to information. Heterogeneity analysis further shows that the effect is stronger among middle-aged and elderly households and in geographically constrained hilly areas. These findings highlight digital connectivity as an important driver of rural energy transitions. From a policy perspective, expanding digital infrastructure, improving digital inclusion, and integrating clean energy promotion with income-enhancing strategies can accelerate the adoption of clean cooking energy and improve rural welfare in developing countries.

## Graphical abstract

**Figure:**
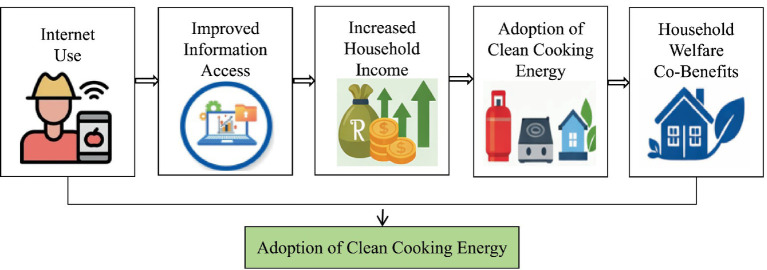

## 1 Introduction

Access to clean and affordable cooking energy is a fundamental prerequisite for improving rural health outcomes and environmental sustainability in developing countries, with potential indirect implications for household food preparation environments and nutrition-related welfare (1–3). Despite notable global progress in expanding energy access, a large share of rural households continues to rely on traditional biomass fuels such as firewood, crop residues, and animal dung for daily cooking. The widespread use of these fuels generates severe indoor air pollution, accelerates environmental degradation, and leads to significant adverse health outcomes, particularly among women and children who experience disproportionate exposure to cooking-related emissions (4, 5).

Beyond direct health risks, dependence on traditional cooking fuels also constrains food safety and dietary quality. Inefficient and polluting cooking technologies limit households’ ability to prepare diverse and nutritious meals under hygienic conditions, thereby undermining the functioning of nutrition-sensitive rural food systems. As a result, clean cooking energy access has increasingly been recognized not only as an energy or environmental issue but also as a critical enabling factor for improved food preparation environments and nutritional well-being.

In Pakistan, the adoption of clean cooking energy remains uneven across rural regions, despite policy initiatives aimed at expanding access to liquefied petroleum gas (LPG), electricity, and other modern energy sources. Rural households, particularly those located in remote and less-developed areas continue to face multiple barriers, including low income levels, inadequate infrastructure, weak market integration, and limited access to information regarding clean energy technologies (6–8). These constraints slow household-level energy transitions and have broader implications for rural welfare, food safety, and nutrition outcomes.

A substantial body of literature has examined the determinants of household energy choices, highlighting the roles of income, education, household characteristics, fuel prices, and policy interventions (9–12). Micro-level evidence consistently shows that economic capacity is a key determinant of clean energy adoption, as modern cooking fuels often involve higher upfront and recurrent costs than traditional biomass fuels (13). Other studies emphasize the importance of behavioral factors, environmental awareness, and access to information in shaping household energy decisions (14).

At the same time, rapid expansion of digital infrastructure has transformed information access and economic opportunities in rural areas (15). Internet use and artificial intelligence have emerged as an increasingly important driver of behavioral change by enabling households to access information on health risks, environmental issues, market opportunities, and public services (16, 17). Empirical evidence suggests that internet use promotes the adoption of green technologies, enhances environmental awareness, and increases household income by reducing information asymmetries and transaction costs (18–21).

From a broader rural welfare perspective, digital connectivity may influence clean cooking energy adoption through both informational and economic pathways. Internet access can raise awareness of the health and environmental benefits of clean cooking technologies, while internet-enabled income opportunities may relax financial constraints that prevent households from switching to cleaner fuels. Cleaner cooking environments, in turn, are associated with improved food preparation conditions and reduced exposure to indoor air pollution (1, 22, 23). This study draws on diffusion of innovation theory, the information-access framework, and the energy ladder perspective to explain how internet use reduces information asymmetries, enhances economic capacity, and promotes clean energy adoption.

Despite these potential linkages, few empirical studies have explicitly integrated internet use, household income, clean cooking energy adoption, and nutrition-sensitive considerations within a unified analytical framework, particularly in developing-country contexts. Against this background, this study investigates how digital inclusion, through internet use, contributes to sustainable energy transitions by influencing rural households’ adoption of clean cooking energy in Balochistan, Pakistan, using survey data collected in 2025. It further investigates whether household income mediates this relationship by testing a conceptual framework linking digital connectivity, income generation, and household energy transitions.

This study contributes to the literature in three key ways. First, it provides micro-level evidence on the association between digital connectivity and rural household energy transitions in Pakistan using newly collected household survey data. Second, it explores household income as a potential mediating pathway linking internet use and clean cooking energy adoption. Given the cross-sectional nature of the data, the mediation analysis should be interpreted as indicative of plausible mechanisms rather than definitive causal channels. Third, the study highlights the broader welfare implications of clean cooking energy adoption, including improved household health environments and food preparation conditions.

The remainder of this paper is organized as follows. Section 2 presents the theoretical framework and hypotheses, as well as the conceptual framework. Section 3 describes the data sources, variable definitions, and empirical strategy. Section 4 reports the empirical results and robustness checks. Section 5 describes the main findings and discussion, and Section 6 presents the key findings, policy implications, and limitations and future research directions.

## 2 Theoretical framework and hypotheses

### 2.1 Internet use and farmers’ clean cooking energy adoption

Drawing on diffusion of innovation theory and the information-access framework, internet use is expected to facilitate the adoption of clean cooking energy by enhancing information diffusion, reducing uncertainty, and strengthening social learning. Internet use can influence farmers’ adoption of clean cooking energy through multiple and interrelated channels, including improved access to information, expanded social networks, and enhanced economic opportunities. First, digital connectivity increases farmers’ exposure to information related to environmental pollution, health risks associated with traditional biomass fuels, and the availability and benefits of clean cooking energy technologies. Improved access to health- and environment-related information raises risk awareness and strengthens pro-environmental attitudes, thereby increasing households’ willingness to adopt cleaner and safer energy sources for daily cooking (24).

Second, the internet serves as an important platform for social interaction and information exchange, enabling farmers to expand their social networks beyond local communities. Online communication tools and social media platforms facilitate the diffusion of information and strengthen peer effects, which play a critical role in shaping household technology adoption decisions (25, 26). Access to information on clean energy policies, technology options, subsidies, and user experiences reduces uncertainty and perceived adoption risks. By lowering information and transaction costs, internet use increases the likelihood that rural households will transition toward clean cooking energy.

Third, internet use can improve farmers’ participation in labor markets and income-generating activities by enhancing access to employment information, skills training, and non-farm economic opportunities (27–29). Increased engagement in off-farm employment and income diversification helps relax financial constraints that often hinder rural households from investing in cleaner but relatively more expensive cooking energy technologies. From a broader rural development perspective, promoting clean cooking energy through digital pathways can contribute to safer household environments, reduced indoor air pollution, and improved overall well-being (1, 30). Consistent with these theoretical arguments, internet use reduces information asymmetry and accelerates the diffusion of clean energy technologies among rural households. Based on the above theoretical arguments, this study proposes the following hypothesis:

*H1:* Internet use is positively associated with farmers’ adoption of clean cooking energy.

### 2.2 Mediating role of household income in the relationship between internet use and clean cooking energy adoption

In addition to informational mechanisms, this study draws on the energy ladder hypothesis, which posits that households shift from traditional to modern energy sources as their income increases. As a key dimension of rural digitalization, internet use can enhance household income through several complementary mechanisms. First, improved access to labor market information reduces job-search costs and strengthens rural households’ connections to external employment opportunities, thereby increasing participation in non-farm work (27). Second, digital connectivity reduces information and transaction costs in both production and consumption decisions, enabling farmers to make more efficient market choices and improve economic returns (20, 21, 28). Third, internet-enabled entrepreneurial activities including online marketing, service provision, and participation in digital platforms create additional income-generating opportunities for rural households (31).

Household income is a well-established determinant of clean cooking energy adoption. Compared with traditional biomass fuels, clean cooking technologies typically involve higher upfront investments and recurrent operating costs, making affordability a major constraint for low-income rural households. Higher income levels enhance households’ ability to bear these costs and significantly increase the likelihood of adopting modern cooking fuels such as LPG and electricity (32). Thus, consistent with the energy ladder framework, increased income serves as a key enabling factor that facilitates the transition toward cleaner cooking energy.

From a nutrition-sensitive development perspective, income-mediated clean cooking energy adoption can indirectly improve household well-being by reducing exposure to indoor air pollution and enabling cleaner cooking and food storage practices. These improvements are closely linked to food safety and nutrition outcomes, particularly in rural settings where households experience high exposure to cooking-related health risks (1). By increasing household income, internet use may therefore indirectly promote clean cooking energy adoption and support the development of more sustainable and nutrition-sensitive rural food systems. In addition to the income-mediated pathway, internet use may also influence clean cooking energy adoption through behavioral and informational mechanisms. Improved access to digital information can enhance households’ awareness of the health risks associated with traditional fuels and the benefits of clean energy technologies. It may also reduce uncertainty regarding technology adoption, strengthen environmental awareness, and improve decision-making efficiency. These complementary pathways suggest that internet use affects household energy choices through both economic and non-economic channels. Accordingly, this study proposes the following hypothesis:

*H2:* Household income mediates the relationship between internet use and farmers’ clean cooking energy adoption.

### 2.3 Conceptual framework

**Figure 1:**
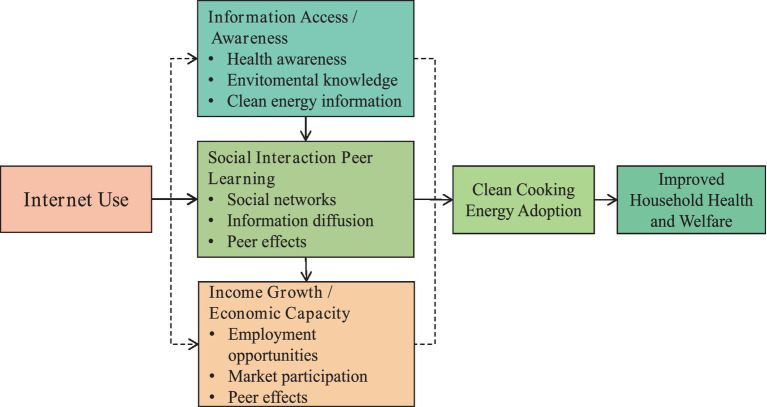
Conceptual Framework.

Figure 1 illustrates the conceptual framework guiding this study and summarizes the theoretical mechanisms through which internet use influences rural households’ adoption of clean cooking energy in Pakistan. Internet use is conceptualized as the core explanatory variable and affects clean cooking energy adoption through two primary pathways: an economic pathway and an informational pathway. These pathways are informed by the diffusion of innovation theory, the information-access framework, and the energy ladder hypothesis.

The economic pathway operates through household income, which serves as a mediating variable. Internet use enhances households’ access to income-generating opportunities by reducing information asymmetries, expanding employment options, and facilitating participation in digital markets. Increased income relaxes financial constraints associated with the adoption of cleaner cooking fuels, such as liquefied petroleum gas (LPG) and electricity. This reflects the income-driven transition emphasized in the energy ladder hypothesis.

The informational pathway reflects improved access to information, awareness, and knowledge regarding the health, environmental, and economic benefits of clean cooking energy. By reducing uncertainty and perceived risks associated with new technologies, internet use directly increases households’ willingness to transition away from traditional biomass fuels. This is consistent with diffusion of innovation and information-access mechanisms. Clean cooking energy adoption, in turn, leads to improved cooking conditions, reduced indoor air pollution, and safer food preparation environments, generating important health and nutrition-related co-benefits. Together, these pathways provide the theoretical foundation for the empirical analysis examining the direct effect of internet use, the mediating role of household income, and heterogeneity in clean cooking energy adoption across rural households.

## 3 Methodology

### 3.1 Study area and data sources

The data used in this study were collected through a household survey conducted by the research team in rural areas of Balochistan Province, Pakistan, in 2025. Balochistan, located in southwestern Pakistan, was selected as the study area due to its predominantly rural population, geographical remoteness, and persistent challenges related to energy access and household welfare. These characteristics make the province particularly suitable for examining clean energy adoption and its implications for rural food systems and nutrition-sensitive development.

**Figure 2:**
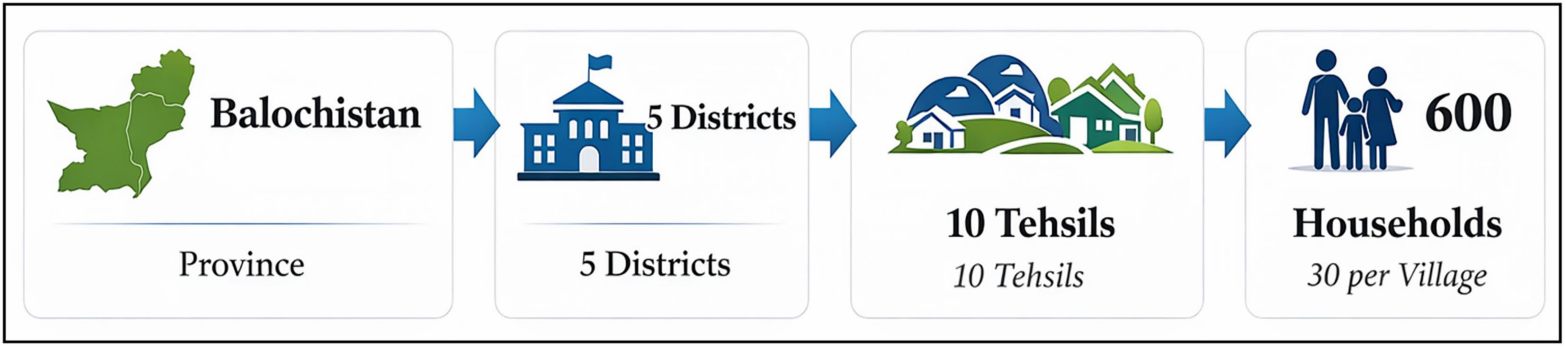
Household survey sampling design.

A stratified random sampling approach was employed to ensure representative coverage across administrative and geographic units. Specifically, sampling was conducted at the district, tehsil, and village levels. The final sample comprised 600 farm households drawn from 5 districts, 10 tehsils, and 20 villages (Figure 2). Households were randomly selected within each village from updated village household lists. To ensure data quality and analytical consistency, observations with incomplete or ambiguous responses regarding household energy use were excluded. After data cleaning, a total of 600 valid household observations were retained for the empirical analysis. The sample size of 600 households ensures sufficient statistical power and representativeness for econometric analysis. A multi-stage stratified sampling approach with proportional allocation across districts, tehsils, and villages was applied, with households randomly selected within each stratum. This design captures geographic and socioeconomic diversity while ensuring sufficient observations for robust econometric analysis.

Data collection was carried out through face-to-face interviews using a structured questionnaire administered on a one-to-one basis. This approach helped minimize response bias and ensured the accuracy of reported information. The questionnaire covered three main components. First, household demographic characteristics, including the household head’s age, gender, education level, self-reported health status, household size, and employment characteristics. Second, household income information, including wage income, farm and non-farm business income, and other income sources. Third, household energy use, including the types of fuels used for cooking, such as firewood, crop residues, liquefied petroleum gas (LPG), electricity, natural gas, biogas, and other energy sources.

### 3.2 Research methods

#### 3.2.1 Baseline regression model

Since farmers’ clean cooking energy adoption behavior is a binary choice outcome, this study employs a binary Logit model to estimate the effect of internet use on farmers’ clean energy adoption behavior. Given the binary nature of the dependent variable, the Logit model estimates the probability that a household adopts clean cooking energy. The model is specified as follows in Equation 1:


$$P(Y_{i}=1)=\frac{1}{1+e^{-(\alpha_{0}+\alpha_{1}{\text{Internet}}_{i}+\alpha_{2}X_{i})}}$$ (1)

Where *P*(*Y_i_* = 1) represents the probability that household *i* adopts clean cooking energy.

The baseline econometric specification is given by Equation 2:


$$Y_{i}=\alpha_{0}+\alpha_{1}{\text{Internet}}_{i}+\alpha_{2}X_{i}+\varepsilon_{i}$$ (2)

Where Y*_i_* denotes whether the *i*-th household adopts clean cooking energy (a binary variable); *Internet_i_* represents internet use by household *i*; and *X_i_* is a vector of control variables at both the household and regional levels. The parameter *α*_1_ captures the effect of internet use on clean energy adoption, *α*_0_ is the constant term, and *ε*_i_ is the random error term.

#### 3.2.2 Mediation effect model

To empirically test the mediating role of household income in the relationship between internet use and farmers’ clean energy adoption behavior and to verify Hypothesis 2, this study adopts the mediation effect analysis framework proposed by Hu et al. (33). The mediation model consists of three equations, specified as follows:

First, the total effect of internet use on clean cooking energy adoption is estimated:


$${\text{CookingEnergy}}_{i}=\alpha_{0}+\alpha_{1}{\text{Internet}}_{i}+\alpha_{2}X_{i}+\mu_{i}$$ (3)

Second, the effect of internet use on the mediating variable (household income) is examined:


$${\text{Income}}_{i}=\beta_{0}+\beta_{1}{\text{Internet}}_{i}+\beta_{2}\;X_{i}+\mu_{i}$$ (4)

Third, clean energy adoption is regressed on both internet use and household income:


$${\text{CookingEnergy}}_{i}=\delta_{0}+\delta_{1}{\text{Internet}}_{i}+\delta_{2}{\text{Income}}_{i}+\alpha_{3}X_{i}+\mu_{i}$$ (5)

In these equations, *Internet_i_* is the core explanatory variable, *CookingEnergy_i_* is the dependent variable representing clean cooking-energy adoption by household *i*, and *Income_i_* is the mediating variable capturing household income. Parameters *α*_1_, *β*_1_, *δ*_1_, *δ*_2_ are estimated coefficients, and *μ*_1_, *μ*_2_, *μ*_3_ are random error terms.

Equation 3 estimates the total effect of internet use on clean cooking energy adoption (*α*_1_). Equation 4 measures the effect of internet use on household income. Equation 5 examines both the direct effect of internet use (*δ*_1_) and the indirect effect through household income (*δ*_2_). If *δ*_2_ is significant and *δ*_1_ < α_1_, it indicates that household income partially mediates the relationship between internet use and clean cooking energy adoption.

#### 3.2.3 Endogeneity and instrumental variable strategy

Potential endogeneity in the relationship between internet use and clean cooking energy adoption may arise from reverse causality, omitted variables, or self-selection. To address this issue, this study employs an instrumental variable (IV) approach. A valid instrument must satisfy two conditions: relevance (correlation with internet use) and exogeneity (affecting clean cooking energy adoption only through internet use). Household broadband installation is used as the instrument. It satisfies the relevance condition as it is a prerequisite for internet access, and the exclusion restriction as it reflects infrastructure availability rather than household energy preferences. To further mitigate potential bias, the model controls for household income, demographic characteristics, geographic factors (altitude and terrain), and regional fixed effects. The model is estimated using the Conditional Mixed Process (CMP) estimator, which accounts for potential correlation between error terms.

### 3.3 Variable selection

#### 3.3.1 Dependent variable

The dependent variable is clean cooking energy adoption, defined based on the household’s primary cooking fuel. It is measured using the survey question: “What fuel does your household mainly use for cooking?” Households reporting LPG, electricity, natural gas, or biogas as their main cooking fuel are coded as 1, while those primarily using firewood, crop residues, animal dung, coal, or other traditional fuels are coded as 0. This classification is based on the dominant fuel used for daily cooking activities. Although fuel stacking is common in rural areas, households are categorized according to their primary cooking fuel to reflect their main energy choice.

#### 3.3.2 Core independent variable

The key explanatory variable is internet use, measured by the survey question: “Does any household member use the internet?” Responses are coded as 1 if at least one household member reports using the internet and 0 otherwise. This binary indicator captures household-level access to digital connectivity, which may influence information acquisition, income opportunities, and technology adoption decisions.

#### 3.3.3 Mediating variable

The mediating variable is per capita household income, calculated as total annual household income divided by the number of household members. Total household income includes wage income, farm income, non-farm business income, and other income sources. To reduce potential skewness and improve the normality of the distribution, the income variable is transformed using the natural logarithm of per capita household income measured in Pakistani Rupees (PKR).

#### 3.3.4 Control variables

To account for confounding factors that may influence household energy choices, several control variables are included at the individual, household, and village levels. At the individual level, the controls include the household head’s gender (male = 1, female = 0), age (years), education (years of formal schooling), and self-rated health status (1–10 scale). These characteristics may affect energy preferences through differences in human capital, awareness, and decision-making capacity. At the household level, family size reflects the number of household members and captures differences in consumption needs and cooking energy demand. Dietary diversity (DietDiv), measured as the number of food groups consumed (0–12), is included to reflect household consumption patterns and socioeconomic conditions. Social insurance coverage (Security) indicates whether the household head is enrolled in any formal social protection program (e.g., health insurance or pension schemes). Social insurance may enhance financial security and reduce budget constraints, potentially affecting the household’s ability to adopt modern cooking fuels. Although descriptive statistics show no significant difference in Security between adopters and non-adopters, it is retained to control for socioeconomic heterogeneity. At the village level, altitude (meters) and terrain type are included to capture geographic and infrastructural differences that may affect access to clean cooking energy. Terrain types include hills, mountains, plateau, plains, grassland, and other categories. Detailed definitions of all variables are presented in Table 1.

**Table 1:** Variables definition.

Variable	Definition
Dependent variable	
Fuel	Household adopts clean cooking energy (1 = yes; 0 = no)
Independent variable	
Internet	At least one household member uses the internet (1 = yes; 0 = no)
Mediating variable	
Income	Natural logarithm of per capita household income (PKR/year)
Individual-level variables	
Gender	Gender of the household head (male = 1; female = 0)
Age	Age of the household head (years)
Education	Years of formal schooling of the household head
Health status	Self-rated health status of the household head (1–10; higher values indicate better health)
Household-level controls	
DietDiv	Household dietary diversity score (0–12 food groups)
Security	Whether the household head is covered by social insurance (1 = yes; 0 = no)
Family size	Number of household members
Village-level controls	
Altitude	Altitude of the village (meters)
Landscape	Terrain type (1 = hills, 2 = mountains, 3 = plateau, 4 = plains, 5 = grassland, 6 = other)
Sample size	600 households

## 4 Results

### 4.1 Descriptive statistics of key variables

Table 2 presents the descriptive statistics of the key variables used in the analysis. Clean cooking energy adoption (Fuel) is a binary variable with a mean value of 0.398, indicating that approximately 39.8% of households use clean cooking energy, while the majority still rely on traditional fuels. Internet use is also measured as a binary indicator, with 56.3% of households reporting access to the internet. Household income shows considerable variation across the sample, reflecting income heterogeneity among rural households. Regarding individual characteristics, approximately 45% of household heads are male, and the average age is 44.65 years. Educational attainment is relatively low, with an average of 2.38 years of schooling, indicating limited human capital among rural residents. The average self-rated health status is 6.62 on a scale of 1 to 10, suggesting moderate health conditions. At the household level, the average household size is 2.80 members, while the social security index averages 11.66, indicating variation in social protection across households. Dietary diversity (DietDiv) has a mean value of 5.52 food groups, suggesting moderate dietary diversity among rural households. Village-level characteristics also vary considerably. The average altitude is approximately 518 meters above sea level, reflecting geographical heterogeneity across the study area, while the landscape variable indicates differences in terrain conditions across villages. Overall, the descriptive statistics demonstrate sufficient variation in the key variables, providing a suitable basis for subsequent econometric analysis.

**Table 2:** Descriptive statistics of key variables.

Variable	Mean	SD	Min	Max
Fuel	0.398	0.488	0	1
Internet	0.563	0.484	0	1
Income	0.931	0.699	0.2	5
Gender	0.450	0.498	0	1
Age	44.65	17.10	17	90
Education	2.379	1.434	0	7
Health status	6.624	1.862	1	10
DietDiv	5.52	1.55	1	10
Security	11.66	4.419	1	18
Family size	2.802	1.067	1	5
Altitude of the village	518.1	302.4	188	1,299
Landscape	1.291	0.828	1	4
Sample size	600			

### 4.2 Benchmark regression results

Table 3 reports the benchmark Logit regression results examining the relationship between internet use and farmers’ clean cooking energy adoption. Column (1) presents the baseline model without additional control variables. The results show that internet use has a positive and statistically significant association with clean cooking energy adoption at the 1% significance level. This finding indicates that households with internet access are more likely to adopt clean cooking fuels. Columns (2)–(4) progressively incorporate individual-, household-, and village-level control variables. Although the magnitude of the internet-use coefficient decreases slightly after the inclusion of control variables, it remains positive and statistically significant at the 1% level across all specifications. This robustness suggests that internet use is an important factor associated with clean cooking energy adoption, thereby providing strong empirical support for Hypothesis H1. This finding is consistent with diffusion of innovation theory and the information-access framework, which emphasize the role of information and communication in technology adoption.

**Table 3:** Internet use and farmers’ clean cooking energy adoption: benchmark regression results.

Variables	(1) Fuel coef.	(1) SE	(2) Fuel coef.	(2) SE	(3) Fuel coef.	(3) SE	(4) Fuel coef.	(4) SE
Internet use	0.715***	0.176	0.629***	0.187	0.630***	0.187	0.597***	0.192
Gender	—	—	−0.271	0.187	−0.276	0.179	−0.316*	0.180
Age	—	—	0.003	0.006	0.003	0.006	0.005	0.007
Education	—	—	−0.014	0.064	−0.014	0.065	−0.020	0.065
Health status	—	—	0.119**	0.052	0.118**	0.050	0.134**	0.061
DietDiv	—	—	—	—	—	—	−0.005	0.036
Security	—	—	—	—	−0.006	0.022	−0.008	0.021
Family size	—	—	—	—	0.039	0.082	0.031	0.084
Altitude	—	—	—	—	—	—	−0.001***	0.000
Mountain area	—	—	—	—	—	—	−0.464	0.420
Plain area	—	—	—	—	—	—	−0.739**	0.350
Constant	−0.828***	0.139	−1.616***	0.504	−1.664***	0.617	−0.794	0.674
Pseudo R^2^	0.0215		0.0322		0.0325		0.0510	
Regional fixed effects	Yes		Yes		Yes		Yes	
Sample size	600							

*, **, and *** denote statistical significance at the 10, 5, and 1% levels, respectively. Robust standard errors are reported in parentheses. All regressions include regional fixed effects. The dependent variable is a binary indicator of whether the household adopts clean cooking energy. Sample size = 600 households.

Among the control variables, self-rated health status shows a consistently positive and statistically significant effect. This suggests that household heads with better health conditions are more likely to adopt clean cooking energy, possibly because healthier individuals have greater awareness of the health risks associated with traditional fuels or possess stronger economic capacity to invest in modern energy technologies. Other individual characteristics, including age and education, do not exhibit statistically significant effects on clean cooking energy adoption. At the household level, social insurance coverage, household size, and dietary diversity are not significantly associated with the adoption of clean cooking energy. At the village level, altitude has a statistically significant negative effect, indicating that households located at higher elevations are less likely to adopt clean cooking energy. This may be attributed to weaker infrastructure, limited market access, and higher transportation costs for modern fuels in remote areas.

Similarly, the negative association observed for mountainous areas may reflect infrastructural and logistical constraints. Although such regions often have easier access to traditional biomass fuels, they typically face higher transportation costs for LPG distribution, weaker electricity infrastructure, and limited market integration. These factors increase the effective cost of adopting modern cooking fuels and may reduce the likelihood of clean energy adoption. Compared with households located in hilly areas, those living in plain areas exhibit a significantly lower probability of adopting clean cooking energy. This finding may reflect regional differences in energy accessibility, infrastructure development, and dependence on traditional fuels. Overall, the benchmark regression results consistently demonstrate that internet use significantly promotes clean cooking energy adoption among rural households.

### 4.3 Robustness checks

To examine the robustness of the benchmark results, this study employs an alternative proxy for internet use. Specifically, the binary indicator capturing general internet access is replaced with a variable measuring whether household members use the internet to obtain weather information. Compared with simple internet access, online weather information use reflects a more active and purpose-driven form of internet engagement, which may better capture households’ effective utilization of digital information. This alternative measure therefore helps address concerns that mere internet access does not necessarily translate into meaningful information acquisition.

In addition, to further alleviate potential concerns regarding sample self-selection into internet use and omitted variable bias, the empirical specification includes a rich set of individual, household, and village-level control variables, as well as regional fixed effects. These controls help reduce bias arising from systematic differences between internet users and non-users based on observable characteristics.

The estimation results are presented in Table 4. Column (1) reproduces the benchmark specification using the general internet-use variable, while Column (2) reports the results based on the alternative measure of internet use related to online weather information. After controlling for individual-, household-, and village-level characteristics, as well as regional fixed effects, the results indicate that internet-based access to weather information has a positive and statistically significant effect on clean cooking energy adoption at the 1% level. The magnitude of the estimated coefficient is similar to that obtained in the benchmark regression, suggesting that the effect of internet use on clean cooking energy adoption remains stable under the alternative specification. Overall, the robustness test confirms that the positive association between internet use and clean cooking energy adoption is not sensitive to the specific measurement of internet use. These findings provide further support for the reliability of the benchmark results.

**Table 4:** Impact of internet use on farmers’ clean cooking energy adoption: robustness test.

Variables	(1) Fuel	(2) Fuel
Internet use	0.597*** (0.191)	
Online weather information		0.635*** (0.193)
Control variables	Yes	Yes
Regional characteristics	Yes	Yes
Pseudo R^2^	0.0510	0.0549
Sample size	600	

*** denotes statistical significance at the 1% level. Robust standard errors are reported in parentheses.

### 4.4 Endogeneity test

Potential endogeneity in the relationship between internet use and clean cooking energy adoption may arise from selection bias, reverse causality, or omitted variables. For example, households with stronger environmental awareness or better unobserved economic conditions may be more likely to both use the internet and adopt clean energy. To address this concern, we employ an instrumental variable (IV) approach using household broadband installation as an instrument for internet use. Broadband installation strongly predicts internet access, satisfying the relevance condition. Regarding the exclusion restriction, broadband availability may correlate with infrastructure development or household economic capacity. However, the empirical specification controls for per capita household income, demographic characteristics, altitude, terrain type, and regional fixed effects, which capture observable economic and infrastructural differences. Conditional on these controls, broadband installation is unlikely to directly influence clean cooking energy adoption other than through facilitating internet use. Thus, the instrument can be considered plausibly exogenous.

The model is estimated using the Conditional Mixed Process (CMP) estimator, which allows joint estimation of equations with different dependent variable types while accounting for correlated error terms. The CMP results, reported in Table 5, show that broadband installation has a positive and statistically significant effect on internet use in the first stage, confirming instrument relevance. The estimated correlation parameter (atanhrho_12) is statistically significant at the 1% level, indicating the presence of endogeneity and supporting the use of the IV approach. After correcting for endogeneity, internet use remains positively and significantly associated with clean cooking energy adoption, with a larger coefficient than in the benchmark Logit model. This result further supports the theoretical expectation that digital connectivity facilitates technology adoption through information and behavioral channels. This suggests that baseline estimates may underestimate the true effect. Overall, the IV results reinforce the robustness of the main findings.

**Table 5:** Impact of internet use on farmers’ clean cooking energy adoption: robustness test.

Variables	cmp_cont (internet use) coef.	SE	cmp_oprobit (clean energy adoption) coef.	SE
Household broadband installed	0.498***	—	—	—
Internet use	—	—	1.319***	—
Control variables	Yes		Yes	
lnsig_1	−0.917***	0.030	—	—
atanhrho_12	−0.581***	0.103	—	—
Sample size	600			

*** denotes statistical significance at the 1% level. Robust standard errors are reported in parentheses. Control variables include individual-, household-, and village-level characteristics. All regressions include regional fixed effects.

### 4.5 Mediating effect of income between internet use and farmers’ clean cooking energy adoption

This study further examines whether household income mediates the relationship between internet use and clean cooking energy adoption using a stepwise regression framework. The estimation results are reported in Table 6, controlling for individual-, household-, and village-level characteristics, as well as regional fixed effects. Column (1) presents the baseline model, confirming that internet use significantly increases the likelihood of adopting clean cooking energy. Column (2) reports the results of the income equation, showing that internet use significantly increases per capita household income. This finding is consistent with the energy ladder hypothesis, which links higher income to the adoption of modern energy sources. This finding suggests that internet access may enhance households’ economic opportunities by facilitating information access, improving market participation, and expanding employment options. Column (3) includes both internet use and household income in the clean energy adoption equation. The results indicate that household income has a positive and statistically significant effect on clean cooking energy adoption. Meanwhile, the coefficient of internet use decreases compared with Column (1) but remains statistically significant. The reduction in the magnitude of the internet-use coefficient suggests the presence of partial mediation, indicating that internet use affects clean cooking energy adoption both directly and indirectly through its impact on household income.

**Table 6:** Mediating effect of income between internet use and farmers’ clean cooking energy adoption.

Variables	(1) Fuel coef.	SE	(2) Income coef.	SE	(3) Fuel coef.	SE
Internet use	0.587***	0.189	0.355***	0.050	0.401**	0.202
Gender	−0.316*	0.178	−0.032	0.046	−0.301*	0.184
Age	0.005	0.007	0.003*	0.002	0.004	0.007
Education	−0.022	0.066	0.015	0.017	−0.028	0.067
Health status	0.134**	0.051	0.034***	0.014	0.116**	0.052
DietDiv	0.026	0.015	0.037	0.014	0.016	0.013
Security	−0.010	0.019	0.006	0.006	−0.012	0.022
Family size	0.031	0.082	−0.386***	0.020	0.262**	0.110
Altitude	−0.002***	0.000	0.000	0.000	−0.002***	0.000
Income	—	—	—	—	0.590***	0.179
Mountain area	−0.450	0.4200	−0.104	0.108	−0.409	0.419
Plain area	−0.739**	0.3550	−0.059	0.087	−0.726**	0.342
Constant	−0.794	0.674	1.381***	0.168	−1.625**	0.722
R^2^	—		0.433		—	
Pseudo R^2^	0.05210		—		0.0660	
Regional fixed effects	Yes		Yes		Yes	
Sample size	600					

*, **, and *** denote statistical significance at the 10, 5, and 1% levels, respectively. Robust standard errors are reported in separate columns.

To further verify the mediating effect, bootstrap tests with 1,000 replications are conducted. The results confirm that the indirect effect of internet use on clean cooking energy adoption through household income is statistically significant at the 95% confidence level, providing empirical support for Hypothesis H2. It should be noted that the analysis is based on cross-sectional data, which does not capture the dynamic timing of income changes. The economic benefits associated with internet use may accumulate gradually rather than occur immediately. Therefore, the mediation results should be interpreted as reflecting contemporaneous associations that may embody sustained or prior internet engagement, rather than short-term income changes caused by current internet use.

### 4.6 Heterogeneity analysis

To examine whether the association between internet use and clean cooking energy adoption varies across different contexts, this study conducts subgroup analyses based on household head age and regional topography. The results are reported in Table 7, including estimated coefficients and subsample sizes. Across age groups, internet use exhibits a positive and statistically significant effect among middle-aged households (*N* = 247) and elderly households (*N* = 171), whereas the effect is not statistically significant among younger households (*N* = 182). The relatively larger coefficient observed for elderly-headed households should not be interpreted as evidence of higher digital literacy among older individuals. Instead, internet use is measured at the household level, and in many rural settings, younger household members are more likely to access and utilize digital technologies. Information acquired through these channels may then be transmitted to the household head, who typically retains decision-making authority over major investments, including cooking technologies. Therefore, the stronger association observed among elderly-headed households likely reflects intra-household information diffusion rather than direct internet use by older individuals.

**Table 7:** Heterogeneous effects of internet use on households’ clean energy adoption by age group and topography.

Variables	Young	Middle-aged	Elderly	Plains	Hilly areas	Mountainous
Internet use	0.400 (0.398)	0.680* (0.364)	0.761* (0.399)	0.203 (0.609)	0.630*** (0.209)	1.399 (0.939)
Control variables	Yes	Yes	Yes	Yes	Yes	Yes
Sample size	182	247	171	138	312	150

*, and *** denote statistical significance at the 10% levels, respectively. Robust standard errors are reported in parentheses. All regressions control for individual, household, and village-level characteristics. Subsample sizes are reported to enhance transparency regarding statistical power in subgroup analysis.

With respect to regional heterogeneity, the results indicate that the positive association between internet use and clean cooking energy adoption is statistically significant in hilly areas (*N* = 312), but not in plains (*N* = 138) or mountainous areas (*N* = 150). One possible explanation is that hilly regions face greater information asymmetries and higher transaction costs in accessing clean energy technologies. Under such conditions, internet access may generate larger marginal benefits by reducing uncertainty regarding fuel availability, subsidy programs, and supplier access. Although physical infrastructure constraints remain relevant, digital connectivity may partially alleviate informational barriers, thereby producing stronger observed effects in these areas. It should be noted, however, that the smaller subsample sizes for plains and mountainous regions may reduce statistical power, and thus non-significant results should be interpreted with caution rather than as definitive evidence of no effect.

To further explore heterogeneity across economic conditions, this study considers the role of household income within the broader analytical framework. Rather than conducting separate subgroup regressions by income level which may lead to reduced statistical power due to smaller subsample sizes the analysis incorporates income as a mediating variable, as reported in Table 6. The results demonstrate that internet use significantly increases household income, which in turn promotes the adoption of clean cooking energy. This finding suggests that the effectiveness of internet use in facilitating clean energy transitions is conditioned by households’ economic capacity. In particular, households with greater financial resources are better positioned to translate improved information access into actual adoption of modern cooking technologies, whereas financially constrained households may face barriers despite having access to digital information. Overall, the heterogeneity analysis highlights that the impact of internet use on clean cooking energy adoption is context-dependent, varying across demographic, geographic, and economic dimensions. These findings underscore the importance of considering both structural constraints and enabling factors such as income and information access when designing policies aimed at promoting clean energy adoption in rural areas. However, given the limitations associated with subsample sizes and cross-sectional data, these results should be interpreted as indicative rather than conclusive.

## 5 Discussion

This study provides robust micro-level evidence that internet use is positively associated with clean cooking energy adoption among rural households in Pakistan. Across multiple empirical strategies including benchmark Logit models, robustness checks using alternative internet-use measures, and instrumental variable estimation the results consistently indicate that households with internet access are more likely to transition from traditional biomass fuels to cleaner cooking energy sources. These findings extend the household energy literature by identifying digital connectivity as an increasingly important, yet underexplored, determinant of clean cooking energy adoption in rural and resource-constrained settings.

The positive relationship between internet use and clean cooking energy adoption can be explained through both informational and economic channels. First, internet access enhances households’ exposure to information regarding the health risks of traditional cooking fuels, environmental pollution, and the benefits of clean energy technologies (34). Improved access to such information can increase health awareness and environmental consciousness, thereby strengthening households’ willingness to adopt cleaner cooking practices. This mechanism aligns with previous studies emphasizing the role of information access and awareness in shaping pro-environmental and health-related behaviors in rural areas (14, 16, 35).

Second, the mediation analysis reveals that household income partially mediates the relationship between internet use and clean cooking energy adoption. Internet use significantly increases per capita household income, which in turn improves the affordability of cleaner but relatively more expensive cooking fuels such as LPG and electricity. This is also consistent with the employment-related characteristics captured in the survey, as internet use may enhance access to labor market information and non-farm employment opportunities, thereby supporting income growth. This finding aligns with the energy ladder and energy stacking hypotheses, which emphasize economic capacity as a central constraint in household energy transitions (12). The partial mediation effect further suggests that internet use influences energy choices not only by enhancing income but also through direct channels such as information acquisition and behavioral change. These findings provide direct empirical support for the theoretical framework outlined earlier, particularly the diffusion of innovation perspective, by showing that internet use accelerates information diffusion and reduces uncertainty associated with adopting clean cooking technologies. At the same time, the results reinforce the information-access framework by demonstrating that digital connectivity influences household decision-making through both knowledge enhancement and income-mediated pathways, thereby extending traditional models of technology adoption in rural contexts.

From a broader rural welfare perspective, these findings have important implications. Although this study does not directly estimate nutritional or health outcomes, clean cooking energy adoption is closely linked to safer food preparation environments, reduced indoor air pollution, and improved household conditions for cooking and food preparation. These factors are critical components of nutrition-sensitive food systems, particularly in rural contexts where women and children face high exposure to cooking-related health risks (1, 23). Household dietary diversity is included as a control variable to account for variation in household consumption patterns and socioeconomic conditions that may influence energy choices.

The heterogeneity results further provide insights into the mechanisms underlying the observed relationships. The stronger association observed among elderly-headed households does not imply higher digital literacy among older individuals. Internet use is measured at the household level, and younger household members may access online information that is subsequently shared with elderly household heads, who typically make key energy decisions. In this way, internet use may strengthen intra-household information transmission and indirectly influence decisions made by elderly household heads. Similarly, the stronger effects observed in hilly areas may reflect higher marginal returns to information in geographically constrained regions. Such areas often face greater information asymmetries and higher transaction costs. Internet access can reduce these informational barriers regarding clean energy technologies and related policies, thereby generating relatively larger impacts despite existing physical infrastructure constraints. Overall, these findings contribute to the growing literature on digitalization, energy transition, and rural development by demonstrating that internet use can serve as a complementary policy lever for promoting clean cooking energy adoption. Policies that integrate digital infrastructure expansion with clean energy promotion and income-enhancing interventions are likely to generate synergistic benefits for public health, environmental sustainability, and nutrition-sensitive food systems in rural Pakistan and similar developing-country contexts.

## 6 Conclusions

### 6.1 Key findings

This study examines the role of internet use in promoting clean cooking energy adoption among rural households in Balochistan, Pakistan. Using household-level survey data and multiple econometric strategies including instrumental variable estimation, mediation analysis, and heterogeneity analysis, the results provide robust evidence that internet use is positively associated with clean cooking energy adoption. The findings remain stable across alternative internet-use measures and after addressing potential endogeneity concerns. Further analysis indicates that household income partially mediates this relationship. Internet use enhances income-generating opportunities, which relax financial constraints and improve the affordability of cleaner fuels such as LPG and electricity. At the same time, internet use exerts a direct effect on clean energy adoption, likely through improved access to information, greater awareness of health risks associated with traditional fuels, and reduced uncertainty regarding modern energy technologies. The heterogeneity analysis reveals that these effects are more pronounced among middle-aged and elderly households and in hilly areas, where information asymmetries and market constraints tend to be more severe. From a theoretical perspective, these findings extend the diffusion of innovation and information-access frameworks by demonstrating that digital connectivity influences household energy decisions through both informational and income-mediated pathways. Overall, the findings highlight the important role of digital connectivity in facilitating rural energy transitions and improving household energy choices.

### 6.2 Policy implications

The findings provide several important policy implications. First, digital infrastructure development should be considered a complementary instrument for accelerating clean cooking energy transitions in rural regions. Expanding affordable internet access can enhance information diffusion, increase income opportunities, and indirectly facilitate the adoption of cleaner household energy sources. Second, clean energy promotion policies should be integrated with digital inclusion and rural livelihood programs to address informational and financial barriers simultaneously. Coordinated interventions that combine connectivity expansion with energy-access initiatives are likely to generate stronger impacts than isolated policy efforts. Third, geographically disadvantaged regions particularly hilly and remote areas may benefit disproportionately from improvements in digital connectivity. This suggests the need for targeted and region-specific interventions aimed at reducing information gaps and improving access to clean energy technologies in such areas. In particular, policymakers can leverage digital platforms, mobile-based information services, and targeted financial support (e.g., subsidies or microcredit) to enhance both awareness and affordability of clean cooking technologies among rural households. Although nutrition outcomes are not directly examined in this study, promoting clean cooking energy through digital pathways may contribute to safer food preparation environments, reduced indoor air pollution, and improved household well-being. Integrated strategies linking digitalization, clean energy access, and rural development may therefore generate broader health-related co-benefits.

### 6.3 Limitations and future research directions

This study has several limitations. First, the analysis relies on cross-sectional data, which limits causal inference and prevents clear temporal sequencing among internet use, income, and clean cooking energy adoption. Although an instrumental variable approach is used to reduce endogeneity concerns, the results should mainly be interpreted as associational relationships. Future studies using panel or experimental data could provide stronger causal identification and allow for dynamic analysis of how internet use influences income and energy transitions over time. Second, while the stratified sampling design improves representativeness within the study area, the sample size of 600 households is relatively moderate. Subgroup analyses by age and terrain involve smaller effective samples, which may reduce statistical power. Future research using larger or nationally representative datasets would help validate the robustness and generalizability of the findings.

Third, internet use is measured as a binary household-level indicator and does not capture variations in usage intensity, digital literacy, or specific purposes of internet engagement. This simplified measure may lead to conservative estimates of the true impact of digital connectivity. Future studies should incorporate more detailed indicators of digital engagement. Fourth, clean cooking energy adoption is defined based on the household’s primary cooking fuel. Given the prevalence of fuel stacking in rural areas, this binary measure may not fully capture partial adoption or differences in fuel-use intensity. Future research could use more detailed measures of household energy-use behavior. Finally, although this study discusses broader welfare implications, it does not directly examine nutrition or health outcomes. Future research should explore the potential impacts of clean energy adoption on household nutrition, health, and overall well-being.

## Data Availability

The original contributions presented in the study are included in the article/supplementary material, further inquiries can be directed to the corresponding author.
